# The Beginning of Systems Chemistry

**DOI:** 10.3390/life9010011

**Published:** 2019-01-24

**Authors:** Peter Strazewski

**Affiliations:** Institut de Chimie et Biochimie Moléculaires et Supramoléculaires (Unité Mixte de Recherche 5246), Université de Lyon, Claude Bernard Lyon 1, 43 bvd du 11 Novembre 1918, 69622 Villeurbanne Cedex, France; strazewski@univ-lyon1.fr; Tel.: +33-472-448-234

**Keywords:** population growth, replication, growth order, Darwinian evolution, selection

## Abstract

Systems Chemistry has its roots in the research on the autocatalytic self-replication of biological macromolecules, first of all of synthetic deoxyribonucleic acids. A personal tour through the early works of the founder of Systems Chemistry, and of his first followers, recalls what’s most important in this new era of chemistry: the growth and evolution of compartmented macromolecular populations, when provided with “food” and “fuel” and disposed of “waste”.

## Dedicated to Günter von Kiedrowski, the Founder of Systems Chemistry, on the Occasion of His Retirement

Leslie Eleazer Orgel (1927–2007) was the prophet of Systems Chemistry, his pupil Günter von Kiedrowski is the founder and name inventor of Systems Chemistry, and Eörs Szathmáry is the mastermind of the first theoretical concepts in Systems Chemistry. I am an active witness of Günter’s and Eörs’ first steps in laying the grounds for Systems Chemistry one year before the first workshop on Systems Chemistry took place in Venice, 2005 [1]. So let me give a very short, very personal and subjective view on how Systems Chemistry started. Ever since, the field has evolved in wide steps, but the first questions still remain generally unanswered.

**Figure 1:**
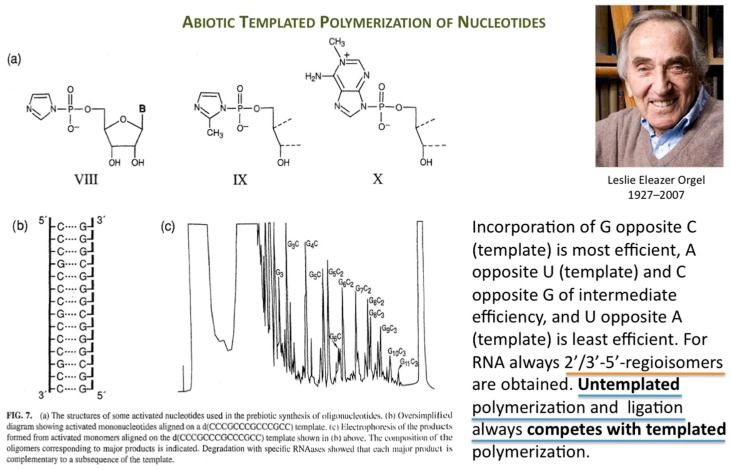
Template-directed enzyme-free RNA chain elongation versus untemplated polymerization and ligation. Parts (a,b,c) taken from [2] and reproduced with permission from Taylor & Francis © 2004.

Orgel’s immense work in prebiotic chemistry and on enzyme-free template-directed nucleic acid chain elongation had a profound influence on the founder of Systems Chemistry (Figure 1).

**Figure 2:**
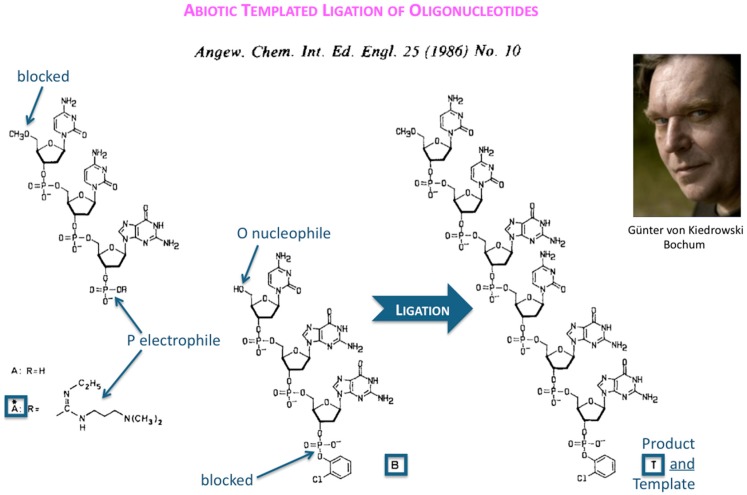
Abiotic templated ligation of oligonucleotides. Structure formula taken from [3] and reproduced with permission from Wiley-VCH Verlag GmbH & Co. KGaA © 1986.

The first success in understanding autocatalytic molecular replicators was pioneered by Günter’s experiments on the enzyme-free autocatalytic chemical fuel-driven ligation of synthetically end-capped DNA fragments A* + B, in particular, the discovery by minute HPLC analysis of the growth rate of these ligated templates T (Figure 2).

**Figure 3:**
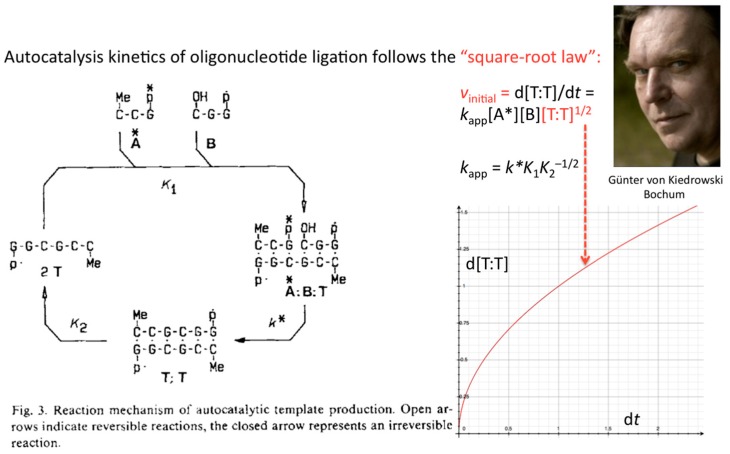
Autocatalytic production of ligation product T from oligodeoxynucleotides A* and B follows a square-root law. Reaction scheme taken from [3] and reproduced with permission from Wiley-VCH Verlag GmbH & Co. KGaA © 1986.

The formulation of an experimentally derived “square-root law” from the fitting of the obtained peak intensities has proven to be a robust concept and general molecular property of self-replicating and cross-replicating macromolecules [4,5] that are in principle able to carry over sequence information through multiple rounds of ligation (Figure 3).

**Figure 4:**
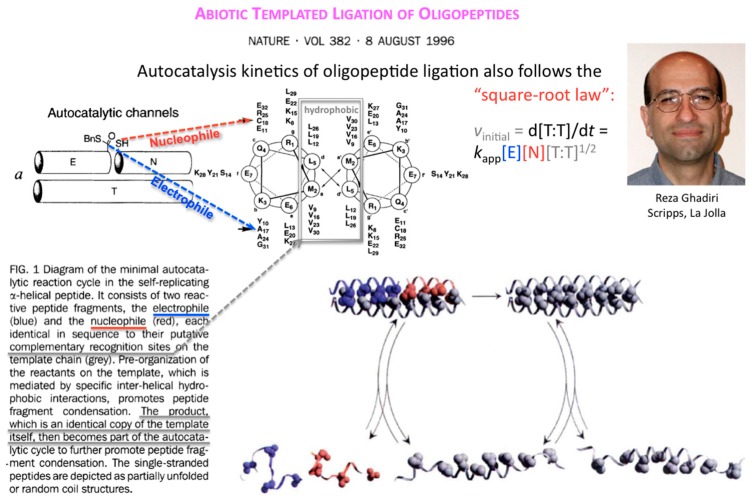
Autocatalytic production of ligation product T from oligopeptides E and N follows the square-root law. Figures and text taken from [6] and reproduced with permission from https://www.nature.com/ © 1996.

Ten years after, Reza Ghadiri and coworkers showed that the square-root law also applies to the kinetics of autocatalytic ligation of synthetically activated peptide fragments, one being electrophilic at its C-terminus (thioester), the other nucleophilic at its N-terminus (Cys thiol) through template-directed native chemical ligation (Figure 4).

**Figure 5:**
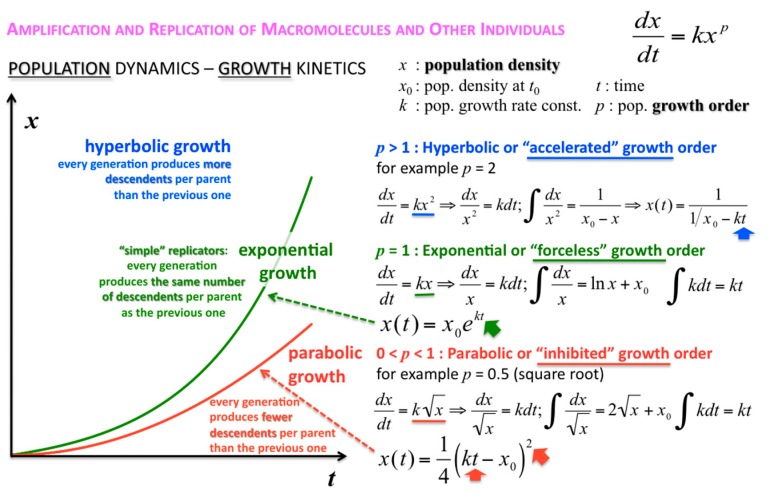
Parabolic (inhibited), exponential (forceless, simple) and hyperbolic (accelerated) growth orders (regimes). Exemplary integrations apply to 1➔2 stoichiometric growth (doublings) only.

The concentration or density of autocatalytic or cross-catalytic molecular—as opposed to supramolecular—replicators in well mixed homogeneous milieus thus grows sub-exponentially with time *t* (Figure 5). For any doubling template population {[T:T] + [T]} = *x*, at any apparent growth rate constant *k*, the resulting parabolic growth order 0 < *p* < 1 describes a growth dynamics where each generation produces on the average fewer descendants per parent than the previous generation (see also right graph in Figure 3). This contrasts exponential and hyperbolic growth orders (*p* ≥ 1) where in each generation, on average, the same number or even more descendants are produced per parent than in the previous generation.

The corresponding growth regimes are termed “inhibited”, “forceless” (“simple”) and “accelerated”, respectively; they apply to all stoichiometries (doubling, tripling and so forth), and explicitly include any selection of the fittest fertile individuals from changes in the environment and the degradation or death rates over time. For example, the human population, domesticated animals and plants—like pigs, cows, chicken, wheat, rice, maize, potatoes, tomatoes, grapes and oranges—globally spread in the accelerated growth regime, owing to increasingly optimised life qualities such as food, fertiliser, health, genetic manipulation, safe transportation and peace. Persisting populations of wild animals and plants, also cloned bacteria and in vitro selected macromolecules (cf. PCR), spread in the forceless growth regime, unless the animals or plants belong to endangered species, the resources are diminishing or the waste is undisposed of for some reason. The inhibited growth regime for the doubling of well-mixed and resourceful autocatalytic and cross-catalytic macromolecules has its roots in a general self-capturing phenomenon termed “strand inhibition”. Without external “help”, usually from enzymes, the unfolding of T:T double-strands (T:T:T triple-strands and so forth, if applicable) is difficult for intrinsic molecular reasons, which is hardly the case for bacterial populations, plants and animals. It is as if grown-up children could not become fully reproductive because, during much of their fertile time, the siblings would prefer to stay together on the playground rather than to go out and mate. Hence, in spite of plentiful resources, fully suppressed side reactions—no degradation or chain elongation instead of replication—and negligible waste product concentrations, viz. under ideal initial conditions, the growth order of the vast majority of macromolecular replicators remains parabolic. The second phenomenal coup out of Günter’s kitchen was to show SPREAD, that is, that the exponential regime can be achieved enzyme-free through the surface-promoted replication and exponential amplification of DNA analogues [7]. The immobilisation of the template strand allows for sequential enzyme-free ligation. The copy is released, and reimmobilised at another part of the solid support to become a template for the next cycle of steps. Irreversible immobilisation of template molecules is thus a means to overcome strand inhibition. In other words, once the grown-up children happen to be out of the playground, don’t let them go back.

**Figure 6:**
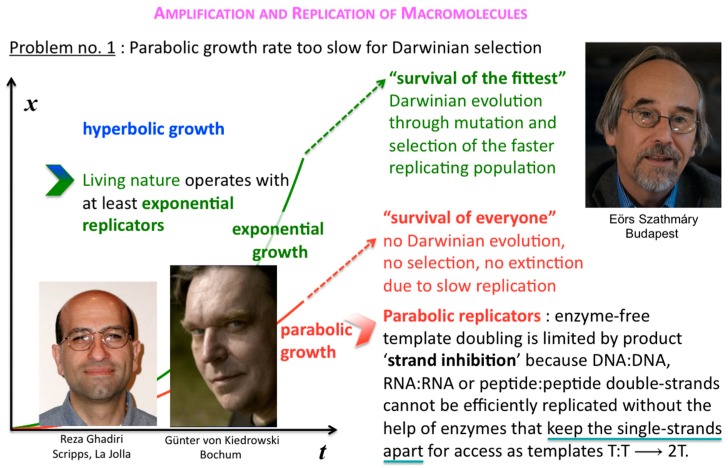
Survival of everyone. Competing but different parabolic replicators (different *k* but same *p*, cf. Figure 5) cannot outcompete one another in a well-mixed milieu.

Before that demonstration, and soon after Günter’s first pioneering discovery, Eörs’ and colleagues’ early insight was to realise that this general strand inhibition was a problem for competing parabolic replicators, and how generally it could be solved [8,9]. In the absence of efficient T:T double-strand unfolders, different macromolecular replicators, bearing markedly different sequences and lengths for example, that are competing for the same resources, can all slowly thrive in the parabolic growth regime, but will virtually never outcompete one another in a well-mixed milieu where food is plentiful and their waste is properly disposed of (Figure 6).

**Figure 7:**
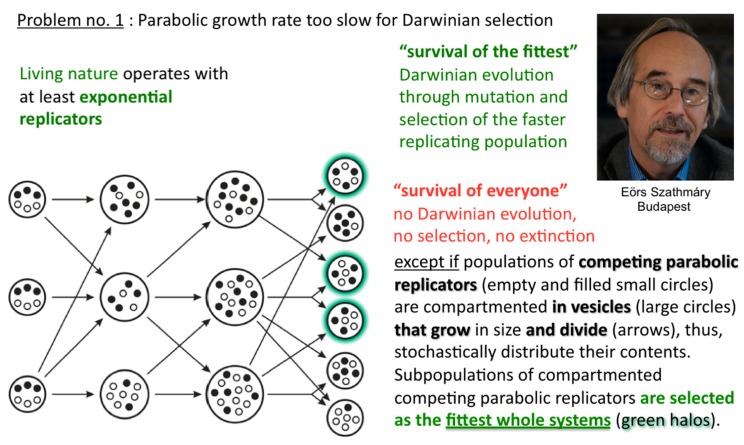
Survival of the fittest whole systems. Once different parabolic replicators are randomly distributed over periodically growing and randomly dividing compartments, the fittest compartments can outcompete less fit compartments, thus, whole populations specify despite the absence of efficient T:T double-strand unfolders.

In such a situation, Darwinian evolution, being defined as evolution through natural selection, as opposed to evolution through genetic drift, migration, mutations, etc., cannot commence. All abiotically produced parabolic replicators will coexist and spread at different rates. In other words, no speciation at the well-mixed macromolecular level is possible. The idea how to solve the problem originates from the notion of group selection. Rather than being well-mixed, compartmented parabolic replicators are in a different population dynamic situation, since selective forces do not affect them directly but address the fitness of whole systems (Figure 7). Eörs calls it the “stochastic (error) corrector” model [10]. This is the most fundamental reason for why life needs to be cellular—other important reasons being confinement, protection, concentration, import-export control, and so forth. My naïve human equivalent: as long as the grown-up children insist on playing instead of mating, those clans that furnish the best housing conditions can maintain their collective fertility potential longer than other clans, who may be at risk of dying without progeny.

**Figure 8:**
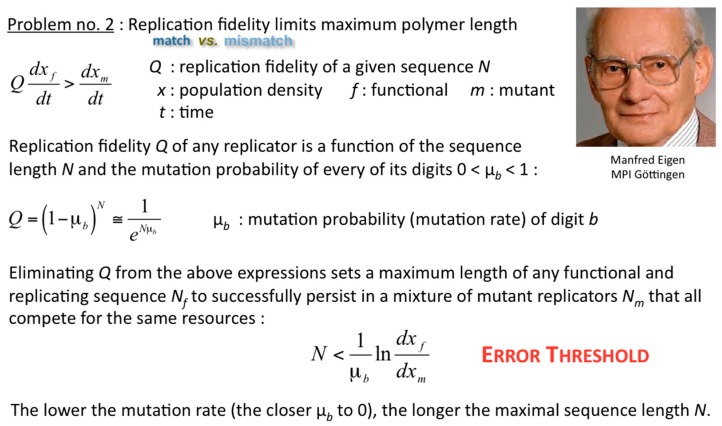
Minimal mutability needed for the robust spreading of useful information. Taken from [12].

Of course, once exponential replicators self-evolve inside selected compartments, the hosting populations are predisposed for their spreading rates to “shoot off exponentially”, if sufficiently fed and disposed of waste products. Such populations can outcompete without hesitation the throng of selected parabolic compartments, now compete with one another in the exponential regime, and spread by Darwinian evolution as we know it from biological cells, organisms and populations. Just how exactly can the integrity of parabolic replicators be maintained long enough throughout their spreading? How can parabolic replicators self-evolve at all? These questions did escort Systems Chemistry right from the start; Eörs exposed yet another fundamental problem that needs to be solved (Figure 8). Manfred Eigen realised long before the founding works of Systems Chemistry that any error propagation sets limits to the amount of information that can be soundly and recurrently inherited through many generations [11]. Solutions to the problem of the self-evolution of the replication fidelity of parabolic, exponential and hyperbolic replicators have been proposed ever since, and are manifold [12], still under vivid debate, and out of the scope of this article.

What can we learn from the pioneering works? The *pudels kern* of Systems Chemistry always was, and still is, the growth and evolution of molecular populations, when provided with “food” and “fuel”, and when disposed of “waste”. Formidable work has been published in the decades that followed this pioneering phase, but there remains much chemistry to be discovered where chemical systems are developed that can “inherit”, i.e., transmit through replication a large amount of highly diverse information (open-ended evolution), that remain robust and dynamically stable over many rounds of replication in the presence of competing replicators and parasites, and that are also sufficiently diverse to be useful for the whole system—therefore, most likely localised in, and carried over from covalent macromolecules—but nevertheless subtly mutable, evolvable, and self-evolvable. This is the essence of Systems Chemistry (to be continued elsewhere).
